# Supplementary material for: Epitaxial Growth of Diamond-Shaped Au1/2Ag1/2CN Nanocrystals on Graphene
Source: Materials (Basel). 2021 Dec 9;14(24):7569. doi: 10.3390/ma14247569 (PMC8706316; doi:10.3390/ma14247569)
Supplement: Supplementary file 1 [file materials-14-07569-s001.zip › materials-1460681 supplementary for conversion.pdf]

Supplementary Materials

Epitaxial Growth of Diamond-Shaped $Au_{1/2}Ag_{1/2}CN$ Nanocrystals on Graphene

Chunggeun Park [1,†], Jimin Ham [1,†], Yun Jung Heo [2,*] and Won Chul Lee [1,*]

[1] Department of Mechanical Engineering, BK21 FOUR ERICA-ACE Center, Hanyang University, 55 Hanyangdaehak-ro, Sangnok-gu, Ansan 15588, Korea; cndrms5568@hanyang.ac.kr (C.P.); jiminham@hanyang.ac.kr (J.H.)

[2] Department of Mechanical Engineering and Integrated Education Institute for Frontier Science & Technology, Kyung Hee University, 1732 Deokyoungdae-ro, Giheung-gu, Yongin 17104, Korea

* Correspondence: wonchullee@hanyang.ac.kr (W.C.L.); yunjheo@khu.ac.kr (Y.J.H.)

† Equally contributed authors.

Captions for Supplementary Videos

Video S1: In-situ TEM movie showing the decomposition process of an $Au_{1/2}Ag_{1/2}CN$ nanocrystal to Au/Ag alloy nanoparticles under electron-beam irradiation. The video plays 60 times faster than real-time. Scale bar: 10 nm.

Video S2: In-situ TEM movie showing the decomposition process of an $Au_{1/2}Ag_{1/2}CN$ nanocrystal to Au/Ag alloy nanoparticles under electron-beam irradiation. The video plays 5 times faster than real-time. Scale bar: 5 nm.

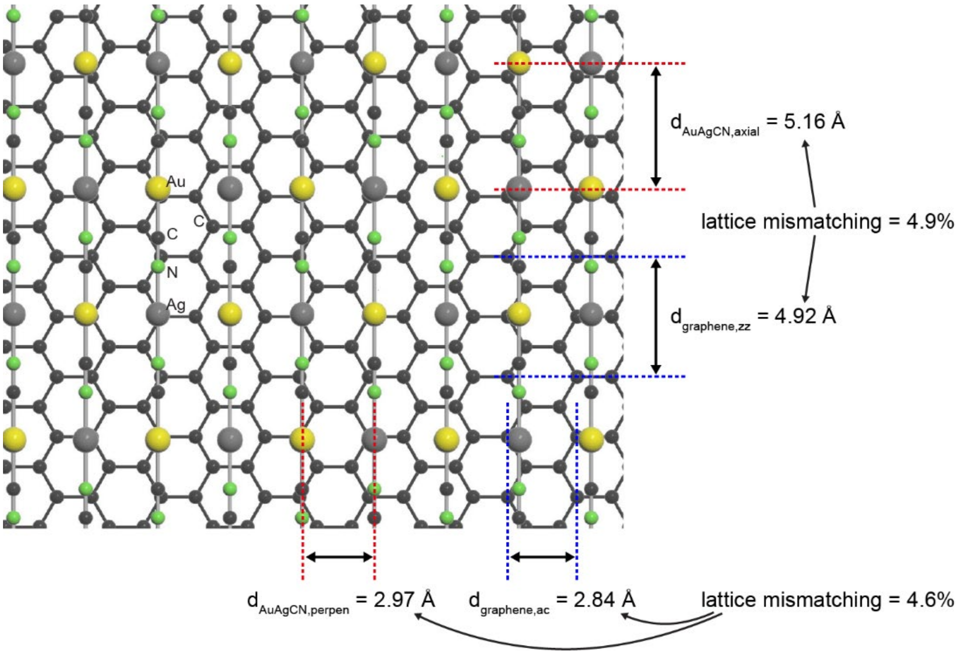

Figure S1. Atomic configuration of $Au_{1/2}Ag_{1/2}CN$ synthesized on graphene. Because van der Waals epitaxy [22,23] is the way the two materials interact with each other, lattice constants of each material are maintained (not adjusted by relaxations) even at the interface. Therefore, there is no short-range periodicity in the atomic arrangement at the $Au_{1/2}Ag_{1/2}CN$-graphene interface.

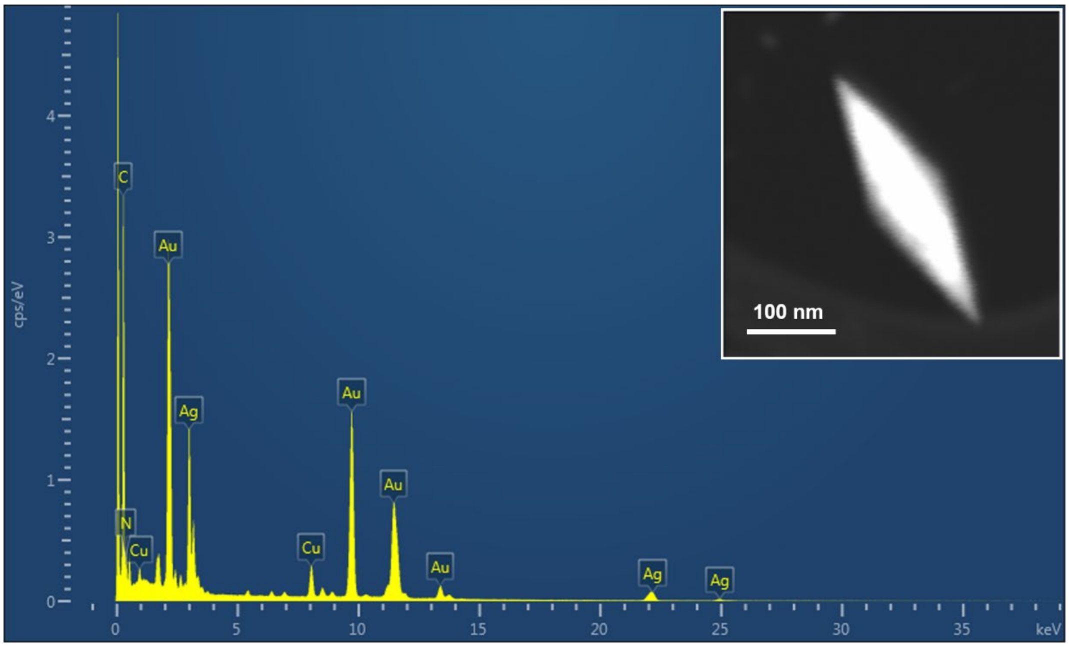

Figure S2. Energy-dispersive X-ray spectroscopic (EDX) analysis of the $Au_{1/2}Ag_{1/2}CN$ nanocrystal synthesized on graphene. The inset shows a darkfield TEM image of the area that the EDX spectrum is measured from. The EDX spectrum clearly indicates that the analyzed sample contains Au, Ag, and C elements. Signals of N and Cu elements might be too weak to give solid evidence for the existence of these elements (N is contained in $Au_{1/2}Ag_{1/2}CN$, and Cu is a common noise signal for this TEM-EDX analysis due to copper sample holders.).

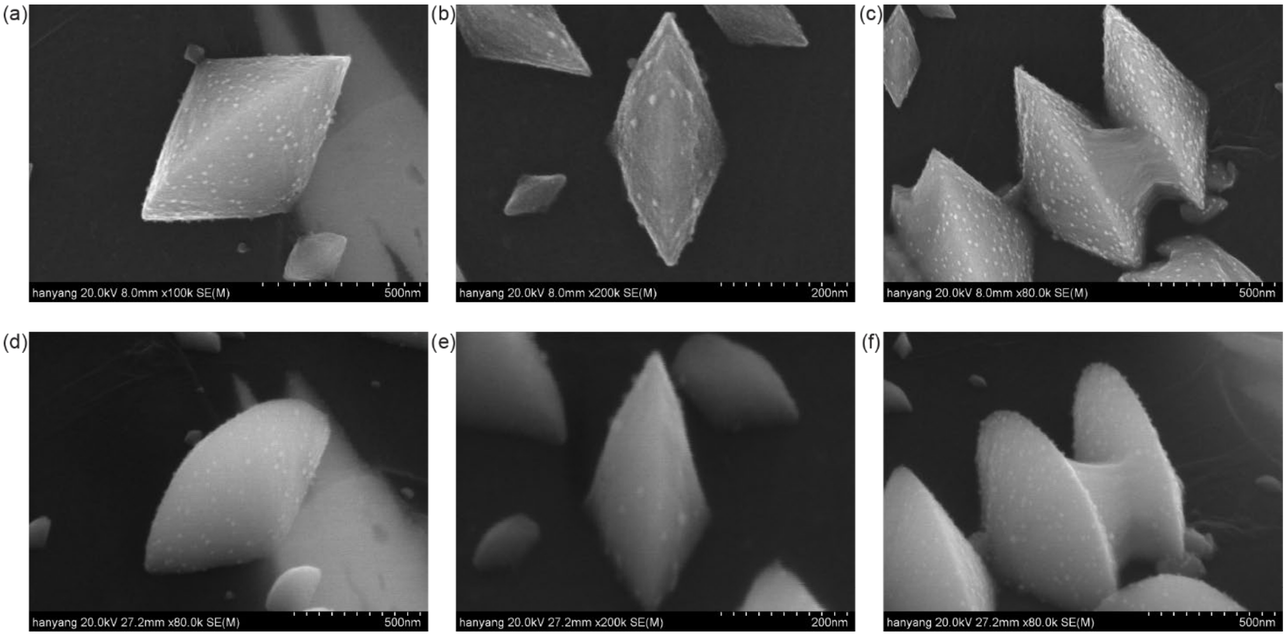

Figure S3. SEM images of the $Au_{1/2}Ag_{1/2}CN$ nanocrystals synthesized on graphene from the top (**a–c**) and with tilting of 40° (**d–f**).
